# Neuromodulation Techniques in Chronic Cerebral Hypoperfusion: Mechanisms and Therapeutic Advances

**DOI:** 10.1002/cns.71089

**Published:** 2026-08-17

**Authors:** Chenye Qiao, Ning Li, Congxiao Wang, Yingpeng Wang, Jinglu Li, Hujun Wang, Zongjian Liu, Shuyan Qie

**Affiliations:** ^1^ Beijing Rehabilitation Hospital Capital Medical University Beijing China

**Keywords:** brain stimulation, chronic cerebral hypoperfusion, cognitive impairment, neuromodulation, synaptic plasticity, vascular cognitive impairment

## Abstract

**Background:**

Chronic cerebral hypoperfusion (CCH) is a major hemodynamic substrate of vascular cognitive impairment and vascular dementia, yet effective therapies with multi‐target neuroprotective potential remain limited. CCH triggers a complex pathological cascade characterized by neurovascular dysfunction, white matter injury, neuroinflammation, oxidative stress, and synaptic impairment.

**Methods:**

In this review, we summarize the major pathophysiological mechanisms of CCH and discuss current evidence supporting neuromodulation as a promising therapeutic strategy. We focus primarily on central stimulation modalities, including repetitive transcranial magnetic stimulation, transcranial electrical stimulation, photobiomodulation, and ultrasound‐based neuromodulation, while also addressing selected peripheral physical modulation approaches and emerging multimodal combination strategies.

**Results:**

Available evidence suggests that these interventions may mitigate hypoperfusion‐related brain injury and cognitive impairment by targeting multiple pathophysiological mechanisms, ultimately restoring synaptic plasticity and promoting circuit‐ and network‐level recovery. We further discuss current challenges to clinical translation, including limited deep‐target engagement, insufficient individualized dosing, and the underuse of rhythm‐based biomarkers.

**Conclusion:**

Neuromodulation holds promise as a mechanistically informed and multi‐target neuroprotective strategy for CCH‐related cognitive impairment.

## Graphical abstract

Chronic cerebral hypoperfusion drives vascular cognitive impairment through neurovascular dysfunction, white matter injury, neuroinflammation, oxidative stress and synaptic network failure. Neuromodulation may offer a multi‐target strategy to restore plasticity, improve functional connectivity and support cognitive recovery.

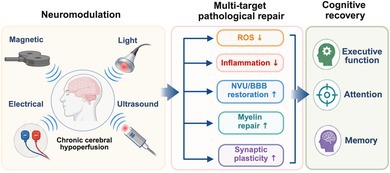

## 1 Introduction

Rapid population aging has increased the global burden of cognitive impairment. Vascular cognitive impairment (VCI) encompasses a continuum from mild cognitive deficits to vascular dementia (VaD), driven largely by cumulative cerebrovascular pathology [1, 2]. Among the major vascular mechanisms, chronic cerebral hypoperfusion (CCH) represents a sustained reduction in cerebral blood flow insufficient to meet metabolic demands and is commonly associated with carotid stenosis or occlusion, cardiac dysfunction, and cerebral small‐vessel disease [3, 4].

CCH is not only the most typical hemodynamic characteristic in the early stages of VCI but also serves as a pivotal pathological hub linking vascular risk factors to structural and functional brain damage [5]. As a persistent and insidious pathophysiological state, CCH drives the progressive deterioration of cognitive function from a compensatory to a decompensated phase by triggering a cascade of events [6, 7], including oxidative stress [8], neuroinflammation [9], blood–brain barrier disruption [10], and impairment of white matter integrity [11]. Consequently, CCH constitutes both the common underlying mechanism for the initiation and progression of VCI and a critical therapeutic target for intercepting the transition from VCI to VaD.

Current clinical management of CCH‐related cognitive impairment remains largely limited to vascular risk‐factor control and symptomatic treatment. Effective therapeutic strategies that directly target neuronal loss, synaptic dysfunction, white matter injury, and circuit‐level impairment are still lacking [12, 13]. Moreover, approaches focused solely on increasing cerebral perfusion may be insufficient, and non‐selective vasodilatory strategies can even worsen perfusion in ischemic territories through intracerebral “steal” phenomena [14, 15].

In this context, interventions capable of modulating neural activity at the network level, engaging endogenous plasticity, and potentially restoring neurovascular homeostasis have attracted increasing attention in CCH‐related translational research [16, 17]. Neuromodulation—using electrical, magnetic, acoustic, or photonic energy—enables relatively safe, controllable regulation of cortical excitability, network connectivity, and oscillatory dynamics [18, 19]. Compared with pharmacotherapies that often act on single molecular targets, neuromodulation may offer multi‐level, convergent regulation across circuits and systems, better matching the network‐level dysfunction that characterizes CCH [20]. In this review, we summarize key pathophysiological cascades and intervention‐relevant targets in CCH, and synthesize current mechanistic and translational evidence supporting neuromodulation as a plausible neuroprotective strategy for VCI/VaD.

## 2 Pathophysiology and Therapeutic Targets in CCH

### 2.1 Hemodynamic Progression and Clinical Phenotype

CCH is a slowly progressive ischemic state characterized by a transition from early hemodynamic compensation to eventual failure of cerebrovascular and metabolic reserve [21, 22]. In the early phase, compensatory vasodilation and collateral recruitment through the Circle of Willis and leptomeningeal anastomoses may partially preserve CBF, leaving pathological changes clinically silent. With persistent hypoperfusion, increased oxygen extraction fraction gives rise to “misery perfusion,” while distal watershed white‐matter tracts and fronto‐subcortical circuits become particularly vulnerable, often manifesting as executive dysfunction [23, 24, 25].

As autoregulatory and metabolic reserves decline, injury extends beyond white matter to gray‐matter structures, including the hippocampus, leading to neuronal loss and disrupted structural and functional connectivity [22]. Clinically, this stage is associated with a broader cognitive profile involving learning and memory deficits, often accompanied by neuropsychiatric symptoms such as apathy and depression [3]. Together, these changes reflect a transition from mild cognitive impairment to VaD along an increasingly refractory disease course.

**FIGURE 1:**
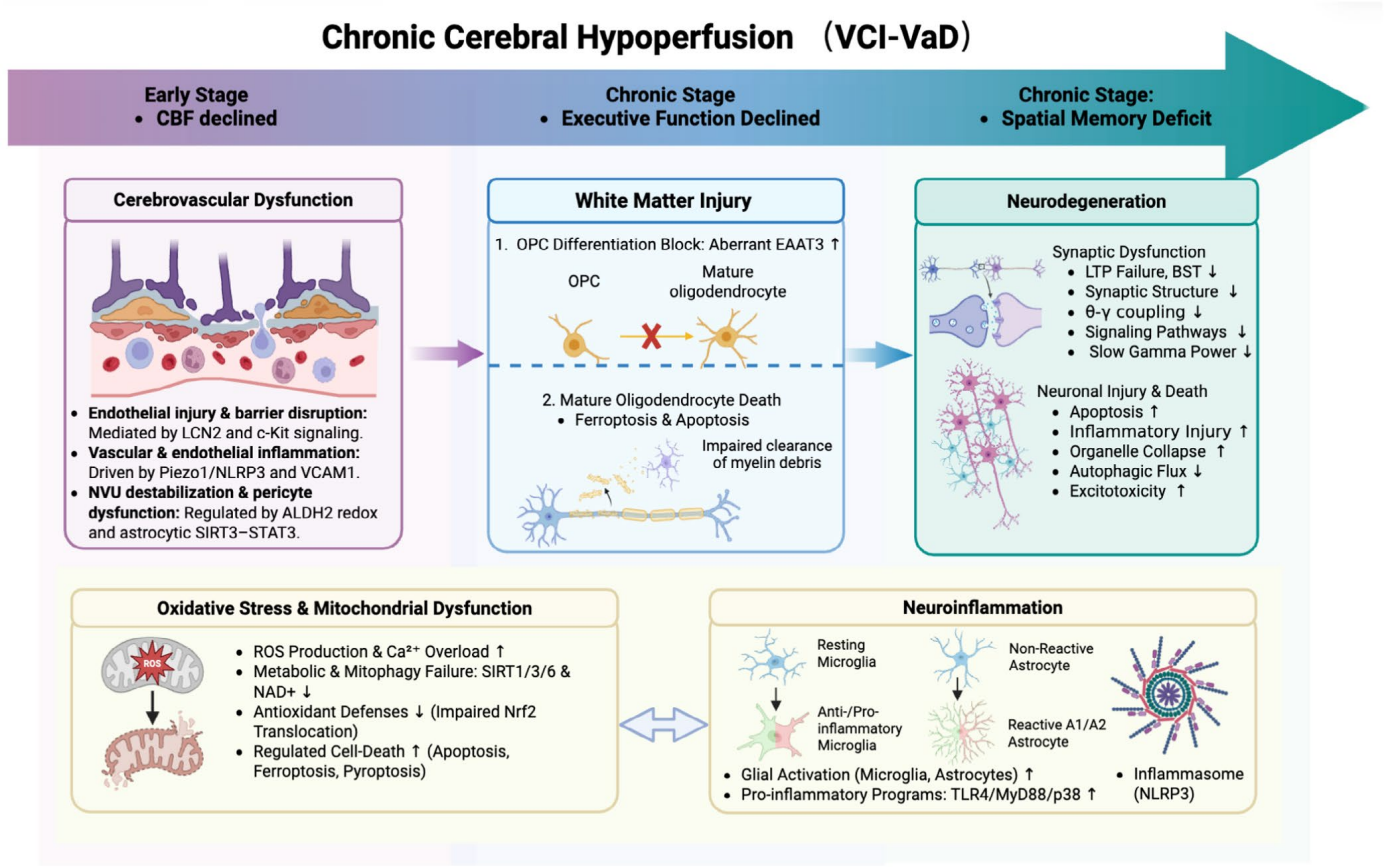
Schematic overview of the multistage cascade of chronic cerebral hypoperfusion leading to vascular cognitive impairment (VCI) and progression to vascular dementia (VaD). ALDH2, aldehyde dehydrogenase 2; BST, basal synaptic transmission; CBF, cerebral blood flow; c‐Kit, tyrosine‐protein kinase KIT; EAAT3, excitatory amino acid transporter 3; LCN2, lipocalin‐2; LTP, long‐term potentiation; NAD+, nicotinamide adenine dinucleotide; NLRP3, NOD‐like receptor family pyrin domain containing 3; Nrf2, nuclear factor erythroid 2‐related factor 2; NVU, neurovascular unit; OPC, oligodendrocyte precursor cell; ROS, reactive oxygen species; SIRT, sirtuin; STAT3, signal transducer and activator of transcription 3; VCAM1, vascular cell adhesion molecule 1; VCI‐VaD, vascular cognitive impairment‐vascular dementia; *θ*–*γ* coupling, theta–gamma phase‐amplitude coupling.

CCH drives VCI through a stage‐dependent but highly interconnected cascade involving neurovascular dysfunction, white matter injury, neuroinflammation, oxidative–mitochondrial stress, synaptic failure, and network disruption. Figure 1 summarizes this multistage cascade and highlights the pathological modules relevant to the neuromodulation strategies discussed below.

### 2.2 Cerebrovascular Dysfunction

Disruption of blood–brain barrier (BBB) integrity and neurovascular unit (NVU) dysfunction are key upstream events linking CCH to VCI [4, 26]. BBB impairment may precede overt cognitive decline and accelerate white‐matter microstructural damage, thereby priming downstream neurodegenerative processes [1]. Vascular comorbidities, particularly chronic hypertension, further reduce cerebrovascular reserve and adaptive capacity to sustained hypoperfusion, hastening cognitive deterioration [27].

At the cellular level, endothelial injury, pericyte dysfunction, endothelial inflammatory responses, and impaired neurovascular coupling collectively destabilize the BBB/NVU interface. Representative mechanisms include LCN2‐ and c‐Kit‐related endothelial barrier disruption, Piezo1/NLRP3‐ and VCAM1‐mediated vascular inflammation, ALDH2‐related redox regulation, and astrocytic SIRT3–STAT3 signaling [10, 28, 29, 30, 31, 32, 33, 34, 35, 36, 37]. From a therapeutic perspective, these mechanisms point to intervention‐accessible targets, including CBF regulation, BBB/NVU stabilization, vascular inflammation control, and restoration of neurovascular coupling.

### 2.3 White Matter Injury and Demyelination

White matter injury is a major structural consequence of chronic hypoperfusion and an important substrate for multidomain cognitive impairment. Deep white matter is particularly vulnerable to chronic hypoperfusion due to its location in distal watershed territories [21]. This persistent ischemic stress triggers widespread damage, characterized by neuroinflammatory activation, oligodendrocyte dysfunction, and progressive tract degeneration [38]. Consistently, clinical neuroimaging in Moyamoya disease indicates that subclinical myelin disruption may precede overt structural lesions and correlate with cognitive decline [39].

At the cellular level, maladaptive oligodendrocyte‐lineage responses are central to white‐matter injury and failed repair; for example, aberrant EAAT3 upregulation can suppress oligodendrocyte precursor cell (OPC) proliferation and differentiation [40]. Sustained neuroinflammatory activation and impaired microglial clearance of myelin debris disrupt OPC differentiation, leading to progressive myelin loss and structural disconnection [41, 42, 43, 44, 45]. These large‐scale tract disruptions and maladaptive neuroinflammatory states represent clinically relevant targets for non‐invasive neuromodulation strategies aimed at supporting remyelination and restoring functional connectivity.

### 2.4 Neuroinflammation

Within the chronic ischemic milieu, persistently activated microglia and astrocytes establish self‐reinforcing inflammatory circuits that promote neuronal apoptosis, synaptic dysfunction, and progressive white‐matter damage. Rather than representing a uniform immune response, CCH‐associated neuroinflammation is shaped by glial phenotypic heterogeneity, inflammasome activation, and metabolic stress.

Microglial activation is a central hub in this process. Lineage‐tracing studies suggest that early inflammation is mainly driven by resident microglia rather than infiltrating monocyte‐derived macrophages, with lipid peroxidation–related signals acting as key initiators [46]. Single‐cell RNA sequencing further reveals heterogeneous microglial states in CCH, including a white matter–enriched Cd74‐high subset associated with proinflammatory programs and white‐matter injury progression [47]. At the molecular level, sustained AIM2/NLRP3 inflammasome activation, TLR4/MyD88/p38 MAPK signaling, dysregulated autophagy, and epigenetic or post‐transcriptional regulatory programs can amplify neuroimmune injury under chronic hypoperfusion [48, 49, 50, 51, 52, 53]. For the present review, the key translational implication is that microglial and astrocytic state modulation may serve as a convergent pathological target shared by several neuromodulatory strategies.

### 2.5 Oxidative Stress and Mitochondrial Dysfunction

Mitochondrial dysfunction is an upstream driver of CCH‐related brain injury. Chronic hypoperfusion induces mitochondrial failure, including dysregulated fission–fusion dynamics, impaired mitophagy, Ca^2+^ overload, and excessive ROS production [54, 55], thereby compromising metabolic resilience and amplifying inflammation, regulated cell death, and NVU degeneration.

Sirtuin‐ and NAD^+^‐dependent pathways are central to mitochondrial homeostasis under hypoperfusion. In CCH models, the NAD^+^‐driven sirtuin network, including SIRT1, SIRT3, and SIRT6, is implicated in mitochondrial biogenesis, mitophagy, ROS detoxification, and metabolic resilience [8, 56, 57, 58]. Oxidative stress also acts as a proximal trigger for regulated cell‐death pathways, including apoptosis, ferroptosis, and pyroptosis [59, 60, 61].

From a protective perspective, the Nrf2 pathway represents a central antioxidant defense system; impaired Nrf2 nuclear translocation may limit antioxidant gene activation and exacerbate oxidative injury [62]. These redox‐ and mitochondria‐related pathways constitute intervention‐relevant targets because neuromodulatory approaches may influence oxidative stress, mitochondrial function, and cell‐survival signaling.

### 2.6 Synaptic and Network Dysfunction and Neuronal Loss

Progressive impairment of synaptic plasticity, neuronal viability, and network coordination represents a final common pathway through which CCH culminates in cognitive decline. These terminal phenotypes emerge from convergent upstream insults and are particularly relevant to neuromodulation because stimulation‐based interventions primarily act at the levels of excitability, synaptic plasticity, oscillatory activity, and network communication.

Synaptic plasticity deficits are a key substrate of CCH‐related cognitive impairment, typically manifested as reduced hippocampal long‐term potentiation (LTP) and diminished basal synaptic transmission (BST) [63]. At the structural and molecular levels, CCH disrupts synaptic ultrastructure and reduces synaptic or neuritic markers such as PSD‐95, HOMER1, synaptophysin, and MAP‐2, accompanied by impaired Wnt, cAMP/PKA/CREB, NMDAR/CaMKII, and presynaptic vesicle‐related signaling [64, 65, 66, 67, 68, 69, 70, 71]. Altered cholinergic, GABAergic, and glutamatergic inputs suggest disrupted hippocampal afferent regulation and may be accompanied by a shift in excitatory/inhibitory balance, thereby affecting information coding within hippocampal circuits [72].

At the network level, these synaptic and neurotransmitter abnormalities are reflected by disrupted oscillatory dynamics. CCH weakens hippocampal slow‐gamma power and impairs theta rhythmic activity and theta–slow gamma coupling, forming a functional chain that contributes to learning and memory deficits [72, 73]. Neuronal loss further consolidates these deficits through overlapping mechanisms, including ROS‐driven apoptosis, impaired organelle homeostasis, defective autophagic flux, excitotoxicity, and gut–brain axis‐related inflammatory injury [70, 74, 75, 76, 77, 78, 79, 80]. Together, synaptic failure, network desynchronization, and neuronal loss provide the mechanistic bridge between upstream CCH pathology and cognitive decline and constitute key targets for neuromodulatory intervention.

## 3 Application of Neuromodulation Technologies in CCH

Given the multi‐level pathophysiology of CCH, neuromodulation has emerged as a promising therapeutic approach because it may target disease‐relevant processes. To provide an overview of the current evidence base, Table 1 summarizes preclinical neuromodulation studies in CCH/VCI, including models, stimulation parameters, stimulation positions, pathological targets, and key effects.

**TABLE 1:** Summary of experimental neuromodulation studies in animal models of chronic cerebral hypoperfusion (CCH). This table outlines non‐invasive brain stimulation modalities in CCH models, including stimulation positions, stimulation parameters, pathological targets, and key cognitive, electrophysiological, and molecular effects.

Modality	Intervention	Animal	Disease model	Stimulation position	Stimulation parameters (intensity/time/frequency)	Treatment course	Pathological target	Key effects	References
rTMS	rTMS (HF/LF)	Male Sprague–Dawley (SD) rats (2 months old)	2‐VO	Central point of the sagittal suture (parallel to the parietal bone)	LF‐rTMS: Intensity: 0.5 Tesla, Time: 600 s per session, frequency: 1 Hz; HF‐rTMS: Intensity: 0.5 Tesla, Time: 30 trains per session (20 pulses per train, 2‐s interval between trains), frequency: 5 Hz	Started day 14 post‐surgery, once daily for 10 days	Neuronal survival	Cognitive: improved spatial learning and memory in the MWM. Electrophysiological: not reported. Molecular: increased mTOR and eIF‐4E expression in hippocampal CA1	[81]
	rTMS (LF)	Male SD rats (2 months old)	2‐VO	Central point of the sagittal suture (close to the scalp surface, parallel to the parietal bone)	Intensity: 0.5 Tesla; Time: 600 s per session; frequency: 1 Hz	Began day 14 post‐surgery, once daily for 10 consecutive days	Synaptic dysfunction; apoptosis; synaptic plasticity	Cognitive: improved spatial learning and memory in the MWM. Electrophysiological: enhanced hippocampal CA3‐CA1 LTP. Molecular: increased NMDAR1 and Bcl‐2; decreased Bax	[82]
	rTMS (LF)	Male SD rats (12 weeks old)	2‐VO	Cerebral hemispheres	Intensity: 1.33 Tesla (70% of threshold); Time: 30 pulses per cerebral hemisphere per session; frequency: 0.5 Hz	Started 2 days after surgery, twice a day for 30 consecutive days	Cholinergic dysfunction; BDNF‐related neuroprotection	Cognitive: improved spatial learning and memory in the MWM. Electrophysiological: not reported. Molecular: increased AChE and ChAT activity; increased BDNF immunoreactivity in hippocampal CA1	[83]
	rTMS (LF)	Male SD rats (1–2 months old)	Repeated bilateral carotid clamping with hypotension induction (4 cycles)	Prefrontal cortex	Intensity: 50% of maximum output intensity; Time: 20 min per session, 2‐s inter‐pulse interval; frequency: 1 Hz	5 sessions as one cycle, once every 5 days for a total of 5 cycles	Oxidative stress; apoptosis; synaptic dysfunction; neurotrophic impairment	Cognitive: improved spatial learning and memory in the MWM. Electrophysiological: increased hippocampal PSP amplitude. Molecular: decreased MDA, Bax and Cas‐3; increased GSH, SOD, Bcl‐2, BDNF, GDNF and NMDAR1	[84]
	rTMS (HF)	Male SD rats	Bilateral common carotid artery ligation in two stages (1‐week interval)	Coil perpendicular to the head	Intensity: 80% of the maximum output of the coil; time: 60 trains per session (20 pulses per train, 8‐s interval between trains); frequency: 10 Hz	Started 7 days after surgery, once a day for 5 consecutive days, followed by a 2‐day interval, then another 5 consecutive days (total 10 days)	Neuroinflammation; oxidative stress; ferroptosis	Cognitive: improved spatial learning and memory in the MWM. Electrophysiological: not reported. Molecular: decreased IL‐6, TNF‐α, MDA and Fe^2+^; increased SOD, GSH, Nrf2 and GPx4	[85]
	HF‐rTMS	Male Kunming (KM) mice (15 months old)	Natural aging (no surgical model)	Coil placed 1 cm above the mouse's head	Intensity: 20% maximum output (0.84 T). Time: 14 consecutive days; 10 trains/day; 100 pulses/train; 30 s interval between trains; frequency: 5 Hz or 25 Hz	Once daily for 14 days	Synaptic structural plasticity	Cognitive: improved spatial learning and memory in the MWM, with 5 Hz showing a stronger effect than 25 Hz. Electrophysiological: not reported. Molecular: increased SYN, PSD‐95, BDNF and pCREB; modulated neurotrophin signaling and CREB‐related gene profiles	[86]
	HF‐rTMS	Male C57BL/6J mice (8–10 weeks old)	BCAS	Coil centered on head	20 Hz; 20% intensity; 1 s trains; 14 s inter‐train interval; 20 trains/session; 1600 pulses/day within 20 min	Started 1 day after BCAS； 14 consecutive days (once each morning)	Synaptic dysfunction; white matter injury; apoptosis; neuroinflammation	Cognitive: improved hippocampal learning and memory in the Y‐maze and novel object recognition test. Electrophysiological: not reported. Molecular: increased MAP‐2, synapsin, MBP, BDNF and Bcl‐2; decreased caspase‐3, Bax, IL‐1β, IL‐6 and TNF‐α	[87]
	rTMS (5 Hz)	Male SD rats	2‐VO	Coil over sagittal suture central point; close to scalp; parallel to parietal bone	Intensity: 120% resting motor threshold (RMT). Time: 200 pulses/day (20 trains × 10 pulses; 2 s inter‐train interval); frequency: 5 Hz (0.5 T reported for pulse trains)	Started 1 week post‐model; treated 4 weeks (5 days on, 2 days off per week)	Angiogenesis; synaptic plasticity	Cognitive: improved spatial learning and memory in the MWM. Electrophysiological: enhanced hippocampal CA3‐CA1 LTP. Molecular: increased VEGF, BDNF, NR1 and NR2B; NR2A unchanged	[88]
	rTMS pre‐treatment	Male Wistar rats	2‐VO	Coil center aligned to sagittal suture central point; coil close to scalp and parallel to parietal bone	Intensity: 0.5 Tesla. Time: 20 bunches/day; 10 pulses/bunch; 2–3 s inter‐bunch interval; frequency: 0.5 Hz	Pre‐treatment	Synaptic plasticity	Cognitive: improved spatial learning and memory in the MWM. Electrophysiological: not reported. Molecular: increased BDNF, TrkB and SYN mRNA/protein expression in hippocampal CA1	[89]
	rTMS (LF vs. HF)	Male Wistar rats	2‐VO	Coil centered on sagittal suture central point; close to scalp; parallel to parietal bone; all parts of brain were stimulated	Intensity: 0.5 Tesla. Time: 20 strings/day; 10 pulses/string; 2–3 s inter‐string interval; 5 consecutive days = 1 course; 6 total courses; 2‐day interval between courses; frequency: LF 0.5 Hz; HF 5 Hz (other parameters same as LF)	6 courses (5 days on +2 days off per course cycle)	Synaptic dysfunction; synaptic plasticity	Cognitive: improved spatial learning and memory in the MWM. Electrophysiological: not reported. Molecular: increased BDNF, NMDAR1 and SYN mRNA/protein expression in hippocampal CA1	[90]
	Theta‐burst TMS (iTBS vs. cTBS)	Male C57BL/6J mice	BCAS	Coil centered on head	Intensity: 20% maximum output; max 4.2 T; Time and frequency: 50 Hz triplets repeated at 5 Hz; iTBS: 2 s trains with 8 s inter‐train interval; cTBS: continuous; 600 pulses	Daily from day 14 post‐BCAS for 14 days	White matter injury; Aβ deposition; neuroinflammation; oligodendrocyte dysfunction	Cognitive: attenuated cognitive deterioration in short‐term retention and long‐term spatial memory tests. Electrophysiological: not reported. Molecular: increased Rxrg expression in mature oligodendrocytes	[91]
	Anodal tDCS	Male Sprague–Dawley rats (6–8 weeks)	2‐VO	Active electrode (1.5 cm^2^) on frontal cortex (midpoint between eyes and ears); reference electrode on anterior chest	Intensity: 0.2 mA; Time: 30 min/session; frequency: 0 Hz (direct current)	10 consecutive days (initiated after model establishment/recovery period)	Oxidative stress; neuroinflammation; autophagy; neuronal loss	Cognitive: improved spatial learning and memory in the MWM. Electrophysiological: not reported. Molecular: decreased MDA, ROS, IL‐1β, IL‐6, TNF‐α and LC3‐II/I; increased SOD, GSH and p62	[92]
	Anodal tDCS (two intensities: 75 μA vs. 150 μA)	Male C57BL/6 mice (8 weeks old)	BCCAO	Anode over right frontal cortex (AP + 2 mm, ML + 1.5 mm; 3.14 mm^2^) + cathode on ventral thorax (10 cm^2^)	Intensity: 75 μA (tDCSL) or 150 μA (tDCSH); Time: 30 min/session; frequency: 0 Hz (direct current)	10 sessions over 2 weeks, starting 24 h after surgery	Hippocampal neurogenesis; neuronal injury	Cognitive: improved spatial learning and memory in the MWM. Electrophysiological: not reported. Molecular: increased ephrinb1, EPHB2, MAP‐2 and NMDAR mRNA/protein expression	[93]
tFUS	Transcranial LIPUS	Male WT C57BL/6 mice (2 or 9 months old)	BCAS	Bilateral hippocampus	Intensity: ultrasound pressure 0.51 MPa; time: 5 min/day; frequency: 1 MHz (carrier frequency; 50 ms burst length, 1 Hz repetition frequency, 5% duty cycle)	Starting 24 h post‐BCAS, continued daily for 28 days	Synaptic plasticity; neuroinflammation	Cognitive: improved cognition in the NOR, contextual fear learning, and radial arm water maze tests. Electrophysiological: enhanced hippocampal LTP. Molecular: increased hippocampal Fndc5/irisin levels	[94]
PBM	Photobiomodulation (PBM)	Female SD rats (4 months old)	BCCAO	Frontal cortex	Intensity: 20 mW/cm^2^; Time: 5 min per session; frequency: every other day; wavelength: 808 nm	Started on the day of left common carotid artery ligation, once every other day for 1 month	Hippocampal neurogenesis; glial activation; neuroinflammation	Cognitive: improved spatial learning and memory in the MWM. Electrophysiological: not reported. Molecular: decreased Iba1, NLRP3 and cleaved caspase‐1 expression; ASC unchanged	[95]

Abbreviations: 2VO, two‐vessel occlusion; BBB, blood–brain barrier; BCAS, bilateral common carotid artery stenosis; BCCAO, bilateral common carotid artery occlusion; HF/LF, high−/low‐frequency; iTBS/cTBS, intermittent/continuous theta‐burst stimulation; LTP, long‐term potentiation; MWM, Morris water maze; NVU, neurovascular unit; PBM, photobiomodulation; tDCS, transcranial direct current stimulation; tFUS/LIPUS, low‐intensity pulsed focused ultrasound.

### 3.1 Repetitive Transcranial Magnetic Stimulation (rTMS)

Repetitive transcranial magnetic stimulation (rTMS) is increasingly considered a viable non‐invasive intervention for VCI, particularly in older adults, given its overall safety and tolerability [96]. Systematic reviews and meta‐analyses suggest that rTMS can improve cognitive outcomes in VaD and post‐stroke cognitive impairment [97]. Mechanistically, its benefits are commonly linked to restoration of synaptic plasticity, modulation of neuronal excitability, and circuit‐level reorganization.

#### 3.1.1 Low‐Frequency rTMS:Neuroprotection and Homeostatic Stabilization

Although low‐frequency stimulation (≤ 1 Hz) is generally regarded as inhibitory in the treatment of central nervous system disorders, in ischemic–hypoxic conditions it may exert compensatory and protective effects by dampening pathological excitability and stabilizing the neuronal microenvironment. Repeated 0.5–1 Hz rTMS has been reported to upregulate neurotrophic and synapse‐related factors, including BDNF, GDNF, and NMDAR1, restore synaptic ultrastructure, and facilitate hippocampal long‐term potentiation [82, 84]. It may also partially normalize cholinergic function in the hippocampal CA1 region and reduce oxidative stress–related apoptosis through modulation of the Bcl‐2/Bax balance, caspase‐3 activity, and endogenous antioxidant defenses [83, 84]. Beyond treatment, 0.5 Hz stimulation has also been explored as a preconditioning strategy. rTMS administered prior to the onset of chronic hypoperfusion has been proposed to establish a “primed” neuroprotective state via activation of the BDNF–TrkB–synapsin axis, thereby reducing subsequent cognitive decline [89]. These findings suggest that LF‐rTMS may limit injury amplification while preserving adaptive plasticity in CCH.

#### 3.1.2 High‐Frequency rTMS and Theta‐Burst Stimulation: Plasticity Enhancement and Targeted Repair

Compared with LF‐rTMS, high‐frequency rTMS protocols (≥ 5 Hz) are more consistently associated with enhanced neurotrophic signaling, structural remodeling, and synaptic plasticity [97]. In CCH‐related models, 5 Hz stimulation has been reported to upregulate VEGF and BDNF, modulate NMDAR subunit composition, and facilitate hippocampal LTP recovery [88, 90]. HF‐rTMS may also contribute to white‐matter repair by suppressing proinflammatory glial polarization and increasing myelin‐ and neurite‐related markers, including MBP and MAP‐2 [87]. In addition, 10 Hz stimulation has been linked to reduced hippocampal neuronal ferroptosis through activation of the Nrf2/GPX4 axis [85]. However, higher frequency does not necessarily confer greater therapeutic benefit. For example, 5 Hz stimulation has been reported to outperform 25 Hz in upregulating synaptic structural proteins and activating the BDNF/CREB pathway, suggesting an optimal frequency window for plasticity induction [86].

Despite frequency‐dependent differences in upstream mechanisms, LF‐ and HF‐rTMS may converge on shared endpoints, including improved hippocampal CA1 neuronal morphology, enhanced spatial memory, and increased synaptic protein translation through pathways such as mTOR/eIF4E [81, 90].

Theta‐burst stimulation provides a further example of pattern‐specific neuromodulation. Intermittent TBS has attracted attention because it may extend beyond CBF restoration and promote repair of demyelinating lesions, potentially through Rxrg signaling in mature oligodendrocytes [91]. These findings support a translational strategy in which rTMS frequency and pattern are matched to dominant pathological targets, such as synaptic plasticity enhancement or white‐matter repair, to enable mechanism‐informed personalization.

#### 3.1.3 Clinical Translation of rTMS in Vascular Cognitive Impairment

Clinical evidence increasingly supports the cognitive benefits of rTMS in VCI and related conditions. A meta‐analysis of 20 randomized controlled trials involving 1049 participants reported significant improvements in executive‐function outcomes, commonly assessed using tasks such as the Stroop test and Trail Making Test‐A [20]. Subgroup analyses further suggest that iTBS, higher‐frequency stimulation, longer treatment courses, and combined regimens may produce larger effects in selected populations [20]. Longitudinal studies also indicate that high‐frequency rTMS targeting the dorsolateral prefrontal cortex may induce cognitive gains lasting up to 6 months, potentially accompanied by longer‐term modulation of MMP/TIMP balance [98].

Beyond therapeutic efficacy, TMS may provide neurophysiological markers for patient stratification. When combined with EEG, TMS offers a temporally precise measure of cortical excitability, excitatory–inhibitory balance, and network dynamics across the VCI spectrum [99]. Neurophysiological studies suggest that early‐stage VCI may show increased intracortical facilitation, reflecting compensatory glutamatergic upregulation and differing from the cholinergic‐dominant signatures often seen in neurodegenerative dementias [100, 101]. These TMS‐derived measures may therefore help characterize vascular cognitive impairment, identify high‐risk subgroups, and guide individualized neuromodulation strategies [99].

Overall, clinical rTMS research in CCH/VCI is moving toward mechanism‐informed optimization and personalized treatment. Future priorities include defining stage‐ and target‐specific parameters, aligning protocols with dominant pathologies, and validating TMS‐based biomarkers for response prediction.

### 3.2 Non‐Invasive Electrical Stimulation Technologies

Transcranial electrical stimulation (tES), with transcranial direct current stimulation (tDCS) as a prototypical modality, has gained attention as a non‐invasive intervention for cerebrovascular‐related cognitive dysfunction because of its favorable safety profile and capacity to modulate cortical excitability in a polarity‐ and context‐dependent manner [102]. In CCH models, anodal tDCS has been reported to mitigate neuronal injury through convergent anti‐inflammatory, antioxidant, and plasticity‐enhancing mechanisms. Specifically, tDCS can suppress TLR4/NF‐κB signaling, reduce proinflammatory cytokines, enhance endogenous antioxidant defenses, and lower hippocampal MDA and ROS levels, indicating partial restoration of redox homeostasis [92]. It may also regulate excessive autophagy and NLRP3 inflammasome activation, thereby attenuating ischemia‐related cell injury [103]. In parallel, repeated anodal tDCS can engage ephrinB1/EPHB2/MAP‐2/NMDAR‐related signaling, promoting hippocampal neurogenesis and synapse‐associated protein expression, which may provide structural support for circuit repair and cognitive recovery [93].

Clinical studies further suggest that tDCS may produce target‐ and domain‐specific benefits. In mild VaD, anodal stimulation over the left dorsolateral prefrontal cortex combined with cognitive training has been associated with greater improvements in visual short‐term memory, verbal working memory, executive control, and response speed than training alone [104]. In cerebral small‐vessel disease with gait impairment, longer‐course tDCS has been linked to improved gait speed and stride length, together with increased perfusion in regions such as the orbitofrontal and cingulate cortices, suggesting potential effects on neurovascular coupling and network‐level motor control [105]. tDCS has also been shown in hypoxic stress models to modulate thalamo‐corticolimbic connectivity, with accompanying improvements in alertness, fatigue, and mood [106].

Beyond polarity‐dependent tDCS, frequency‐specific approaches such as transcranial alternating current stimulation (tACS) are of particular interest because they may directly modulate pathological oscillatory dynamics. Although direct evidence in CCH remains limited, studies in Alzheimer's disease suggest that 40 Hz gamma‐frequency tACS can improve cognition by normalizing neural oscillations, enhancing network synchronization, and regulating synaptic plasticity and neuroinflammatory responses [107, 108, 109, 110]. These findings support the potential relevance of rhythm‐targeted electrical stimulation for CCH‐related cognitive impairment, particularly given the oscillatory abnormalities implicated in hippocampal dysfunction.

Although rTMS and tDCS have shown potential for VCI/CCH‐related cognitive impairment, the current clinical evidence remains preliminary and markedly heterogeneous. For rTMS, the primary trials included in the meta‐analysis discussed above commonly showed insufficient reporting of blinding procedures, lack of allocation concealment, and limited use of rigorous sham controls [20]. For tDCS, available evidence in vascular dementia also remains limited; for example, a small single‐blind, sham‐controlled study reported superiority over sham stimulation only in selected cognitive tasks [104]. Therefore, small sample sizes, inconsistent trial quality, differences in control conditions, and the lack of responder analyses may collectively limit the interpretability of current clinical evidence and increase the risk of publication bias. In addition, preclinical CCH models typically involve controlled vascular injury, homogeneous animals, and early standardized interventions, whereas patients with VCI/CCH‐related cognitive impairment often present with mixed pathology, variable cerebrovascular reserve, comorbidities, and heterogeneous cognitive phenotypes. Thus, although preclinical neuroprotective effects support the biological plausibility of neuromodulation, they cannot yet be directly extrapolated to durable clinical efficacy in humans. Larger studies with rigorous sham controls, double‐blind designs, responder analyses, and long‐term follow‐up are still needed.

### 3.3 Photonic and Acoustic Neuromodulation

Beyond magnetic and electrical stimulation, photonic, acoustic, and peripheral neuromodulation approaches provide additional therapeutic avenues for CCH‐related cognitive impairment. Photobiomodulation (PBM) has been explored in both clinical and preclinical settings. Clinically, 610‐nm LED irradiation applied to cervical vertebral and internal carotid artery regions in individuals with mild cognitive impairment increased regional cerebral blood flow in the prefrontal and cingulate cortices, accompanied by improvements in global cognition and memory [111]. In a BCCAO rat model, 808‐nm PBM delivered to the frontal cortex reduced neuroinflammation by inhibiting NLRP3 inflammasome activity and glial activation, while promoting endogenous neural stem cell proliferation and differentiation in the hippocampal dentate gyrus [95].

Ultrasound‐based neuromodulation offers advantages in spatial precision and depth penetration. Low‐intensity pulsed ultrasound has been proposed to exert neuroprotective effects by engaging the hippocampal Fndc5/irisin pathway, enhancing synaptic plasticity, and reshaping the inflammatory microenvironment [94]. Transcranial pulse stimulation, as a specialized high‐pressure acoustic modality distinct from conventional low‐intensity ultrasound, has also been reported to be safe in VCI populations and associated with improved cognitive scores, suggesting a potential non‐invasive option for dementia phenotypes linked to chronic hypoperfusion [112].

**FIGURE 2:**
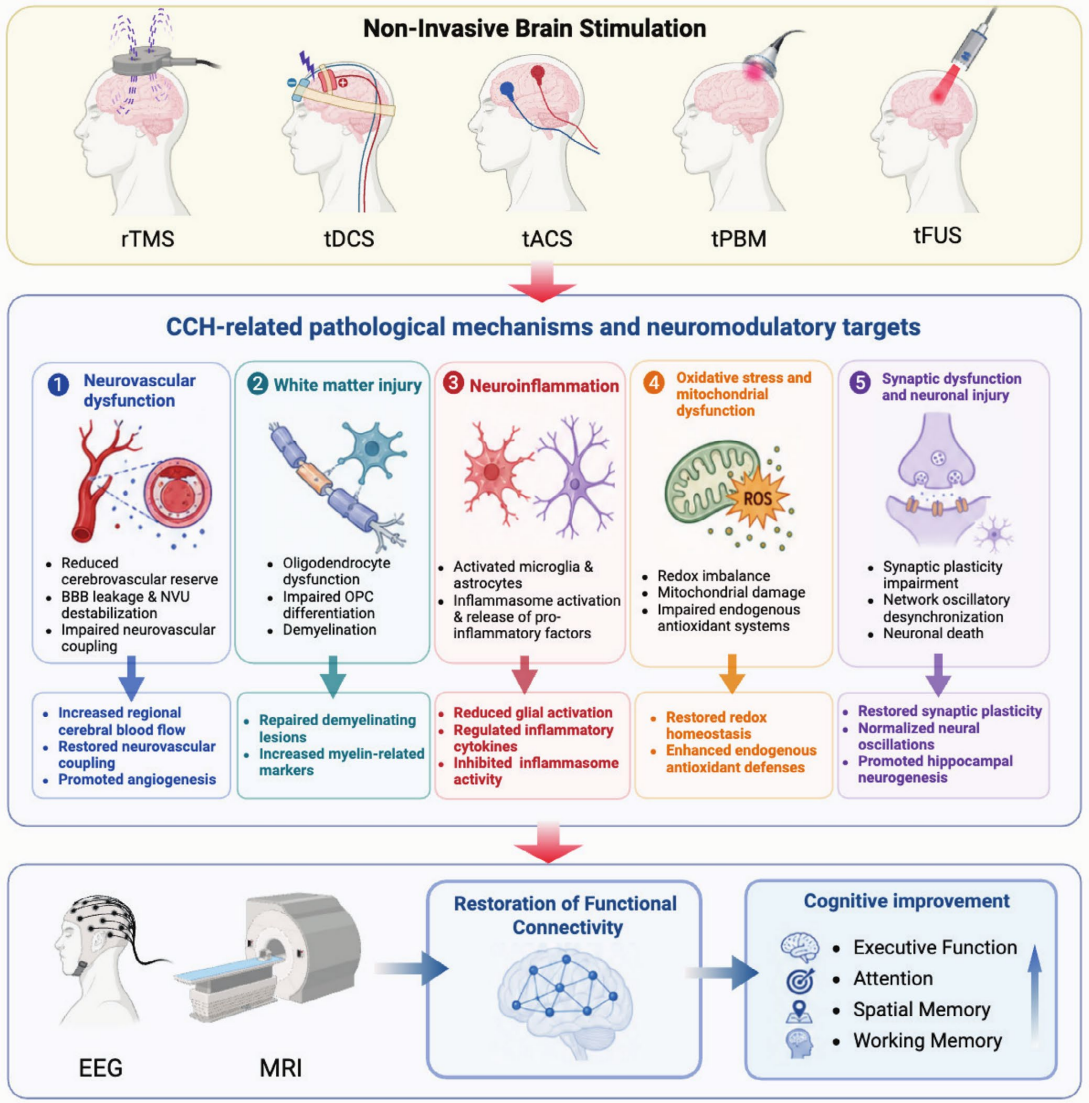
Conceptual framework of pathological mechanisms and therapeutic targets of non‐invasive brain stimulation (NIBS) in chronic cerebral hypoperfusion (CCH). BBB, blood–brain barrier; CCH, chronic cerebral hypoperfusion; EEG, electroencephalography; MRI, magnetic resonance imaging; NVU, neurovascular unit; OPC, oligodendrocyte precursor cell; ROS, reactive oxygen species; rTMS, repetitive transcranial magnetic stimulation; tACS, transcranial alternating current stimulation; tDCS, transcranial direct current stimulation; tFUS, transcranial focused ultrasound; tPBM, transcranial photobiomodulation.

Taken together, the cognitive benefits of NIBS in CCH may be mediated by restoration of synaptic plasticity, improved circuit communication, and large‐scale functional reorganization measurable by EEG and MRI. These effects may ultimately translate into gains in executive function, attention, and memory. This synapse–network–cognition framework is summarized in Figure 2.

### 3.4 Peripheral Neuromodulation and Conditioning Strategies

Beyond direct central stimulation, peripheral conditioning strategies have been explored as potential modifiers of CCH pathology.

VNS and its non‐invasive auricular variant (taVNS) have attracted growing interest because taVNS may modulate astrocytic polarization, reduce BBB permeability, enhance angiogenic responses, attenuate hippocampal neuronal injury, and improve cerebrospinal fluid dynamics [113, 114]. Preliminary clinical work also suggests potential cognitive benefit when taVNS is combined with conventional rehabilitation in post‐ischemic cognitive deficits [115].

Remote ischemic conditioning is another repeatable peripheral stimulation strategy that may act through a “peripheral stimulation–systemic transmission–central benefit” mode. Evidence indicates that RIC can improve cerebral perfusion, preserve NVU integrity, attenuate white matter injury, and suppress inflammation and oxidative stress, with potential cognitive benefits reported in small‐vessel disease‐related MCI and CCH animal models [116, 117, 118, 119]. Overall, these approaches may engage endogenous repair programs through convergent neurovascular, immune, and plasticity‐related pathways, supporting their complementary role in CCH/VCI neuromodulation.

### 3.5 Combined Effects of Neuromodulation and Multimodal Therapies in CCH

Although NIBS and peripheral physical interventions show therapeutic promise, single‐modality approaches may be insufficient to address the multi‐layered pathological network underlying CCH. Emerging evidence suggests that integrating neuromodulation with pharmacotherapy, structured rehabilitation, and adjunct physical therapies may enhance efficacy by engaging complementary neurochemical, neuroimmune, vascular, and circuit‐level mechanisms.

Pharmacotherapy remains a mainstay in VCI, but its ability to directly restore damaged circuits is limited. Clinical studies indicate that rTMS combined with acetylcholinesterase inhibitors, such as donepezil, can produce greater cognitive gains than either treatment alone in mild‐to‐moderate impairment associated with subcortical small‐vessel disease, with larger improvements in global cognitive measures such as MMSE and MoCA [120]. This suggests that electromagnetic modulation of neuroplasticity may amplify drug‐related neurochemical effects by improving the synaptic and network environment.

Combination with rehabilitation may provide another route for synergistic repair. In VaD models, tDCS combined with swimming exercise has shown stronger neuroprotective effects than either intervention alone, potentially through convergent anti‐inflammatory, antioxidant, and metabolic‐regulatory pathways [121].

Clinical exploration is also expanding toward diversified regimens, including rTMS combined with hyperbaric oxygen therapy to address ischemia–hypoxia, electroacupuncture combined with tDCS and computerized cognitive rehabilitation to couple peripheral and central stimulation with task‐driven plasticity, and TMS combined with taVNS to coordinate cortical network modulation with brainstem autonomic engagement [122, 123, 124].

## 4 Limitations and Future Perspectives

Although neuromodulation shows promise for mitigating CCH‐related brain injury, several bottlenecks must be addressed to enable robust clinical translation. First, mainstream NIBS approaches, such as rTMS and tDCS, remain limited by field penetration and spatial focality, with effects largely confined to superficial cortical regions [18]. Consequently, clinical targets often converge on cortical areas such as the dorsolateral prefrontal cortex, whereas direct modulation of deep nodes critical to CCH‐related cognitive phenotypes, particularly hippocampal and limbic circuits, remains challenging. Emerging modalities, including transcranial temporal interference stimulation (tTIS) and transcranial focused ultrasound (tFUS), may help overcome this superficial‐targeting limitation by offering improved depth and spatial specificity.

Second, inter‐individual heterogeneity in VCI may substantially influence neuromodulation responsiveness and treatment selection. Multimodal TMS studies suggest that sex‐related differences are associated with distinct cortical excitability profiles and cognitive trajectories, even under comparable vascular burden [125], potentially reflecting hormonal modulation—particularly by estrogen—of targeted neuroplasticity pathways. However, many NIBS protocols still rely on standardized parameters and insufficiently account for differences in comorbidities, cerebrovascular reserve, and baseline network state. Given the state‐dependent nature of stimulation effects [126, 127], future studies should establish stage‐ and phenotype‐specific therapeutic windows and develop individualized, closed‐loop paradigms integrating imaging/neurophysiological stratification, field modeling, and real‐time EEG or TMS–EEG feedback.

Third, CCH‐related cognitive impairment is frequently accompanied by electrophysiological abnormalities, including reduced hippocampal slow‐gamma power and impaired theta–gamma coupling [72, 128].

Yet current neuromodulation studies often emphasize molecular or structural endpoints, with limited attention to circuit dynamics and cross‐frequency coupling. Rhythm‐targeted approaches, including tACS, tRNS, tTIS, and visual or auditory gamma entrainment using sensory stimulation (GENUS), have shown the capacity to modulate neural oscillations in Alzheimer's disease research [108, 129, 130], but their relevance to CCH‐specific oscillatory abnormalities requires direct validation.

Finally, single‐modality interventions may be insufficient for the multi‐level pathology of CCH [131]. Future work should advance stage‐ and target‐oriented combination strategies that integrate neuromodulation with pharmacotherapy, motor or cognitive rehabilitation, and other physical therapies. Such mechanism‐matched regimens may better engage complementary repair pathways and shift CCH treatment from symptomatic improvement toward mechanism‐based neuroprotection and more durable clinical benefit.

## 5 Conclusion

As an upstream driver of VCI, CCH engages multi‐scale injury cascades that challenge single‐target therapies. By integrating evidence across electrical, magnetic, acoustic, and photonic neuromodulation, this review highlights the therapeutic potential of physical‐energy interventions for CCH. A multi‐target, network‐aware framework may inform mechanistically plausible neuroprotective strategies for VCI/VaD.

## Author Contributions

**Chenye Qiao:** conceptualization, literature analysis, writing – original draft, writing – review and editing; **Ning Li:** methodology, literature screening, writing – review and editing; **Congxiao Wang:** investigation; **Yingpeng Wang:** data curation; **Jinglu Li:** writing – review and editing; **Hujun Wang:** validation; **Zongjian Liu:** methodology, supervision, writing – review and editing; **Shuyan Qie:** conceptualization, supervision, writing – review and editing.

## Funding

This research was funded by National Natural Science Foundation of China (grant number 82302869).

## Consent

The authors have nothing to report.

## Conflicts of Interest

The authors declare no conflicts of interest.

## Data Availability

Data sharing not applicable to this article as no datasets were generated or analyzed during the current study.
